# Dark Triad, Perceptions of Organizational Politics and Counterproductive Work Behaviors: The Moderating Effect of Political Skills

**DOI:** 10.3389/fpsyg.2017.01972

**Published:** 2017-11-08

**Authors:** Muhammad A. Baloch, Fanchen Meng, Zefeng Xu, Ignacio Cepeda-Carrion, Danish, Muhammad W. Bari

**Affiliations:** ^1^School of Management and Economics, Beijing Institute of Technology, Beijing, China; ^2^Department of Business Management, Universidad de Sevilla, Seville, Spain; ^3^Center for Energy and Environmental Policy Research, Beijing Institute of Technology, Beijing, China; ^4^Department of Business Administration, Government College University Faisalabad, Faisalabad, Pakistan

**Keywords:** counterproductive work behavior, Dark Triad, perceptions of organizational politics, political skills, PLS-SEM, hospitality industry

## Abstract

The aim of this work focuses on the relationship among the Dark Triad (psychopathy, narcissism, and Machiavellianism), perceptions of organizational politics, political skills, and counterproductive work behavior. This study empirically tests the mediating role of perceptions of organizational politics in the relationship between the Dark Triad and counterproductive work behavior. Furthermore, the study investigates the moderating role of political skills in strengthening the link between the Dark Triad and the perceptions of organizational politics. A sample of 149 participants was randomly selected. To analyze the data of the present work, we employed a structural equation model using partial least square and PROCESS. From empirical findings, we imply an inference that perception of organizational politics partially mediates the Dark Triad's influence on the counterproductive work behavior. Moreover, the results identify the moderating role of political skills in strengthening the link between the Dark Triad and the perceptions of organizational politics. Empirical findings suggest important policy implications for the hospitality industry.

## Introduction

Counterproductive work behaviors (CWBs) are destructive behaviors for organizations and their members. The magnitude of these activities can range from minor to more severe (Bolton et al., 2010). In recent times, there has been growing interest in explaining and tackling the deviant behaviors of employees at the workplace (MacLane and Walmsley, 2010).

Prior studies have revealed that CWBs result in severe economic and social threats to organizations. Earlier works have reported that global businesses suffered losses of around US$2.9 trillion annually due to fraudulent activities (Berry et al., 2012; Moore et al., 2012). Moreover, the study of Baughman et al. (2012) linked conflict and bullying to CWBs. Berry et al. (2012) identified hyper-anxiety, job dissatisfaction, and tendency to quit a job as common outcomes of CWBs. In a nutshell, CWB is a serious issue for organizations and their stakeholders (Fine et al., 2010).

Many studies have been carried out to find the possible antecedents of CWBs. Mainly, CWBs have been investigated from the perspective of the equity theory proposed by Adams (1966) and theories of aggression (Spector, 1978). The primary focus of these studies was to find the situational or environmental factors of CWBs. For instance, Bowling and Burns (2015) revealed that interpersonal conflict, job satisfaction, and organizational constraints are strongly related to CWBs. In addition, Schwager et al. (2016) showed a significant negative relationship among mindfulness, conscientiousness, and honesty–humility in their hierarchical regression analyses. Karim et al. (2013) indicated that anger is positively related to aggressive CWBs. The stressor-emotion model proposed by Spector and Fox (2005) suggested that negative emotions are important factors of CWB. Furthermore, Fida et al. (2015) found that negative emotions and moral disengagement are strong antecedents of CWB. Recently, Ceschi et al. (2016) observed the mediating role of exhaustion in job demands–CWBs relationship. While honesty–humility positively moderates the relationship between job demands and exhaustion, it also has an adverse effect on the relationship between exhaustion and CWBs.

Previous studies also emphasized the importance of identifying personality characteristics that may relate to CWBs. For example, Berry et al. (2012) and Kozako et al. (2013) investigated the influence of the Big Five personality traits toward CWBs. In this sense, one of the more appealing concepts, one that has received more attention lately, are that the Dark Triad^1^ personalities may be the possible determinants of the development of CWBs (Smith and Lilienfeld, 2013). For instance, Barlett (2016) found that all the Dark Triad traits are associated with aggression. Geel et al. (2017) showed that the Dark Triad traits are related to traditional bullying and cyberbullying behaviors in adolescents and adults. Based on the social exchange theory developed by Emerson (1976), it is found that individuals who score high in the Dark Triad traits are less likely to engage in some types of counterproductive work behavior when there are perceived higher levels of organizational support (Palmer et al., 2017). Similarly, the meta-analysis of O'Boyle et al. (2012) concludes a weak, moderate, and relatively strong relationship of psychopathy, Machiavellianism, and narcissism with CWBs, respectively. Their findings also demonstrated that the overall moderating roles of authority and culture are weak. Grijalva and Newman (2014) reported narcissism as a strong predictor of CWBs among the Dark Triad traits. However, the narcissism–CWB link weakens in the group collectivist culture.

Overall, the relationship between the Dark Triad and CWBs is not clear due to a substantial number of positive, negative, and insignificant findings. However, a mutual consensus is still lacking concerning the relationship between the Dark Triad and CWBs. Cohen (2016) posits that past studies may have ignored some important mediators and moderators in the relationship between the Dark Triad and CWBs. It is possible that the Dark Triad and CWBs' link may be more indirect through other organizational factors than direct. To fill this research gap, the objective of the present work is to investigate the mediating role of the perceptions of organizational politics (POPS) in the relationship between the Dark Triad and CWBs. Moreover, the current study examines the moderating role of political skills in the Dark Triad–POPS relationship.

The present study contributes to the existing literatures in several ways. First, following Cohen's (2016) framework, the present study contributes to social exchange research by empirically testing the mediating role of POPS in the relationship between the Dark Triad and CWBs. Meanwhile, the current study investigates the moderating function of political skill on the Dark Triad and POPS nexus. Second, we extend our research into the hospitality sector of China. CWBs are widespread among hospitality employees and are commonplace in the hospitality industry (Spector, 2010; Spector and Fox, 2010; Jung and Yoon, 2012; Zhao et al., 2013). Therefore, we believe that the hospitality sector is an excellent setting to validate the proposed model.

The study proceeds as follows. The next section states the theoretical background and hypotheses of the study. Section Method describes the research methodology employed to test the hypotheses. Section Results contains the empirical results of the survey. The last section brings together the discussion, conclusion, and future line of research.

## Theoretical background and hypotheses development

### The effect of the dark triad on counterproductive work behavior

Paulhus and Williams (2002) used the term Dark Triad to describe three traits, i.e., psychopathy, narcissism, and Machiavellianism. Although these three personality traits have distinct importance and style, their basic approach is one of apparent and covert exploitation of conspecifics. Each trait depicts a set of substitute and usually a set of condemned interpersonal tendencies, so their relationship to work behaviors is relatively the same (O'Boyle et al., 2012).

According to Wu and Lebreton (2011), deviant behaviors may be the outcome of deviant personality traits. This rationale provides a base for the association between the Dark Triad and CWBs. The lack of accountability and impulsivity of psychopathy, the lack of empathy of narcissism, and the moral degeneration of Machiavellianism work as contributors to CWBs (O'Boyle et al., 2011; Jonason et al., 2012). Wu and Lebreton (2011) have explained why psychopathy relates to CWBs. Those who score high in the traits of psychopathy harm others to pursue their self-interest. They distract others' attention from a particular task and work on their agendas. Furthermore, their conscienceless personality and desire to achieve their goals even at the cost of harming others may lead them to engage in CWBs (O'Boyle et al., 2012). Hare and Neumann (2009) claimed that traits of psychopathy are closely associated with aggressive CWBs. Subsequent studies of Scherer et al. (2013) and Smith and Lilienfeld (2013) reported that psychopathy is related to workplace CWBs.

In the case of narcissism, Spector (2010) contended that inflated self-image is a trait of those who score high in the trait of narcissism. Moreover, narcissistic-type personalities may do whatever is required to exaggerate their selves (Wu and Lebreton, 2011). The cult of self-exaggeration, even putting others down, may increase the possibility of those individuals with narcissistic traits to engage in deviant behavior. It is not surprising that the findings of Penney and Spector (2002) and Grijalva and Newman (2014) showed a positive association between narcissism and CWBs.

With regard to Machiavellianism, Moore et al. (2012) convinced that personalities high in Machiavellianism traits are morally disengaged. They are willing to do whatever is required to attain their goals (Wu and Lebreton, 2011). The ruthless and unethical behaviors of Machiavellian-type personalities provide a strong argument that individuals with these traits are more inclined to exhibit CWBs. Machiavellian characters are also impulsive and irresponsible when engaging in interpersonal interaction. They are less concerned and careless about the consequences of their behaviors (Skinner, 1988). These emotions enhance the possibilities for those personalities to be involved in CWBs. The findings of Kish-Gephart et al. (2010) and O'Boyle et al. (2012) also showed a positive association between Machiavellianism and CWBs.

With regard to the aforementioned arguments, we expect:

H1-a: Psychopathy relates positively to counterproductive work behavior.
H1-b: Narcissism relates positively to counterproductive work behavior.
H1-c: Machiavellianism relates positively to counterproductive work behavior.

### Mediating role of perceptions of organizational politics in the relationship between the dark triad and counterproductive work behavior

Organizational politics is defined as “individual or group behavior that is informal, ostensibly parochial, typically divisive, and above all in a technical sense, illegitimate—sanctioned neither by formal authority, accepted ideology, nor certified expertise (although it may exploit any one of those)” (Ferris et al., 2002). The concept of POPS flourished after the development of the classic model of POPS was proposed by Ferris et al. (1989) and the POPS scale was developed by Kacmar and Ferris (1991). Ferris et al. (1989) proposed that personality serves as an antecedent to POPS. The need–supply perspective of person–organization fit theory states that a fit between a person and an organization occurs when the organization satisfies the individual's need, desire, or preference (Kristof, 1996). The Dark Triad personalities are more motivated to interpret actions and events in political terms because manipulation and opportunism are their personally salient issues. Therefore, the inclinations of the Dark Triad are bent toward a political work environment (Rosen et al., 2006). The study of Valle and Perrewe (2000) also provided sufficient support for the link between the Dark Triad and POPS. Similarly, Yang (2017) further posits that employees perceive their work context as political so they may feel “the rules of the game change as policies, protocol, and conscientious behaviors are replaced with gamesmanship, unwritten norms, and rewards based on influence.”

In the case of the tie between POPS and CWB, the accumulating empirical literature provided a sufficient argument to support the concept that POPS is linked to numerous adverse employee outcomes, i.e., CWB (Chang et al., 2009). Rosen and Levy (2013) state that POPS, as a research issue, is a well-established topic, but more studies are required to clarify how and why POPS provokes deviant behavior from employees. The study of Kacmar et al. (2013) found a negative relationship of POPS with helping and promotability. Similarly, the findings of Cohen and Diamant (2017) also positively related POPS to CWB.

To sum up, the above arguments give ample support to propose that Dark Triad individuals perceive their conditions in a different way than others, and when they perceive the environment as being particularly open to exploitation, they behave in a worse manner. The Dark Triad leads to an increase in POPS, and a higher POPS leads to an increase in CWB. By adding both direct relationships, the current study develops an indirect relationship between the Dark Triad and CWB with a mediating role of POPS. In this way, there is an indirect effect of the Dark Triad on CWB through POPS.

Based on the above arguments, this study thus hypothesizes:

H2-a: Perceived organizational politics positively mediate the relationship between psychopathy and counterproductive work behavior.
H2-b: Perceived organizational politics positively mediate the relationship between narcissism and counterproductive work behavior.
H2-c: Perceived organizational politics positively mediate the relationship between Machiavellianism and counterproductive work behavior.

### The moderating effect of political skills on the relationship between the dark triad and counterproductive work behavior

Organizational politics has always been an important topic of discussion in previous studies. However, the political skills of employees have received less attention in the literature. Individuals having high political skills can settle themselves down in various social situations. They know what to do and how to efface during serving self-motives and pretend to be sincere (Ferris et al., 2005). Political skill is defined as the “ability to effectively understand others at work and to use such knowledge to influence others to act in ways that enhance one's personal and/or organizational objectives” (Treadway et al., 2013). Employees with high political skills know how to serve their egotistic behavior (Ferris et al., 2005). They are able to understand the social context better than others (Treadway et al., 2013). Brouer et al. (2009) considered political skills as a personal resource for employees, which they invest to fulfill their objectives. Politically skilled individuals can adjust their behavior in a way that appears honest and sincere. Furthermore, Bentley et al. (2015) stated that the politically skilled individuals are socially wise; they have the ability to appear natural and make social networks.

The ambiguity in political context also provides an opportunity for politically skilled individuals to execute their unique skills. By using their interpersonal skills, they assess the social nods and align their behaviors with situations (Rosen and Levy, 2013). Politically skilled individuals are also imbued with natural wisdom. They can drive a political environment, have the potential to assess people, and are able to access information through their networks (Rosen and Levy, 2013). In the same way, Kimura (2013) declared that a political climate is a kind of unfair organizational setting where political games influence decisions. Therefore, personalities who rank high in political skills perceive such environments as uncertain and feel fit for them.

Based on the above logic, one can assume that those individuals who score high in the Dark Triad construct have strong political skills and perceive high politics in a work environment because they are conscious of their surroundings. They are able to adjust and calibrate their behaviors to be contextually appropriate. They can disguise their motives to get ahead, and their environment facilitates them to pursue their aims. Nevertheless, the level of political skills does vary among people, including those scoring higher in the Dark Triad construct (Cohen, 2016). For example, Wallace's (2015) contingency model shows that the functionality and dysfunctionality of the traits of narcissism depend on the level of political skills of the individuals high in narcissistic traits. According to Wallace, in the case of ego threat, those with a lot of narcissistic traits who also have strong political skills will adjust their self-maintenance mechanisms more effectively than individuals who have a high level of narcissism but who lack political skills. Similarly, Brouer et al. (2009) posit that individuals who have a lot of Machiavellian traits and are politically skilled have the ability to pretend as to be honest and sincere.

It is evident that the Dark Triad personalities who possess strong political skills are more likely to exploit their political environment. Because of their networking ability and social astuteness, it is easier for them to sense political advantage than those who lack political skills.

Therefore, it can be expected that a relationship between the Dark Triad personalities and perceived organizational politics becomes stronger for those individuals who have strong political skills. Thus, we expect:

H3-a: Political skills positively moderate the relationship between psychopathy and perceived organizational politics.
H3-b: Political skills positively moderate the relationship between narcissism and perceived organizational politics.
H3-c: Political skills positively moderate the relationship between Machiavellianism and perceived organizational politics.

## Method

Partial least squares (PLS) is an appropriate data analysis technique for this study because of the model and sample data characteristics. The PLS technique is based on the structural equation model (SEM) and the measurement model (Henseler, 2017b). We prefer the PLS method over other regression models due to some of its advantages, and it is suitable for the current study for the following reasons. First, it is more robust with fewer identification issues and works with complex research problems. The research model entails considerable complexity concerning the types of relationships in the hypotheses. Second, the sample size of the present study is small (*n* = 149). Third, our measures are composites based on scales designed by researchers (Henseler, 2017a). Fourth, PLS is suitable when measurement models have few indicators (<6) or sample size (>100). As in the current study, most of the indicators are less than six, and the sample size is greater than one hundred, i.e., 149 employees (Hair et al., 2017). Finally, this study not only predicts but also explains the variance among the primarily targeted constructs. The proposed framework and nature of the study is more exploratory than confirmatory. Therefore, we believe that it is a suitable analysis technique for the current research (Carrión et al., 2016). This study employs Smart PLS v.3.2.6 software and the PROCESS macro developed by Hayes (2013) for PLS analysis and moderated mediation analysis, respectively.

### Data collection

The respondents of our study were employees and their supervisors from the hospitality sector of Beijing, a metropolitan city of China. We randomly selected full-time employees as potential participants from the hospitality industry. Members of the research team obtained permission from the HR departments of the respective organizations before collecting data. A letter was submitted to the HR departments of the respective organizations to ensure the confidentiality of the data and the voluntary participation of the respondents. The Ethics Committee at the Beijing Institute of Technology also approved this study.

The data were collected via paper-and-pencil survey, and the participants were asked about the Dark Triad, POPS, political skills, and CWBs. Data were collected during work time with the permission and help of the managers of these organizations. As data were collected from a single source, there is a possibility that common-method bias may arise. As per the guidelines of Podsakoff et al. (2003), we applied the following procedures to minimize this potential issue. First, we provided an introduction at the beginning of the survey to promise confidentiality and to protect the respondents' answers. Moreover, we explained the purpose of the study and ensured them that there were no right or wrong answers and that they should express their opinions as honestly as possible. Second, we developed two different sets of anonymous self-report questionnaires. The first set comprised measures of the Dark Triad, POPS, political skills, and demographic variables, whereas the second set included surveys of CWB.

In addition, we examined the possibility of common-method bias by employing Harman's single factor test. This was conducted by adding all the measurement items into an exploratory factor analysis with an unrotated factor solution. The largest factor only accounted for 48%, indicating that common-method bias was unlikely to be a problem in our survey (Podsakoff et al., 2003). However, it is important to notify that common-method bias cannot be ruled out entirely because all variables were collected as self-report data. Nevertheless, the present study took adequate measures to minimize the possibility of common-method bias. The guarantee of confidentiality and protection of the respondents' information encouraged them to answer the questions as honestly as possible.

We distributed 230 questionnaires to employees in the hospitality sector. In return, 163 questionnaires were retrieved. After scrutiny, we eliminated 14 questionnaires due to improper filling by the respondents and selected 149 for data analysis, resulting in a 65% response rate. Following the guidelines of Cha et al. (2007), we applied the combined technique (back translation and discussion) in translation procedures. A panel of four bilingual experts was hired. Three of them were bilingual experts in the field of organizational science, and one was a professional translator of Chinese to English. The first two bilingual experts from organizational science jointly discussed and translated the survey from the original language to the targeted language, and the translated version was forwarded to a third bilingual specialist for scrutiny. The third translator reviewed the translated version with the first two translators and removed discrepancies and errors. The finalized Chinese version was sent to the professional translator for back translation into English. After comparing both the original and the back-translated English versions, a panel of experts approved the survey instruments in the Chinese language.

The respondents included both males 80 (54%) and females 69 (46%). Forty-five respondents (30%) were between the ages of 18 and 29 years, 72 respondents (48%) were aged between 30 and 40 years, and the rest were older than 40 years. Most of the respondents (84%) had a university education. The majority of those surveyed (65%) had tenure of 5 years or less, whereas the rest had more than 5 years. The majority of respondents [60 (40%)] worked in the front office followed by sales and marketing [37 (25%)], food and beverage [22 (15%)], rooms [20 (13%)], and general management [10 (7%)].

### Variable's measurement

A review of the literature led us to identify measures for each construct that account for validation.

#### Counterproductive work behavior

The study uses an eight-item CWB scale from Dalal and Welch (2009), which has been used in a Chinese context as a measure of CWB (e.g., Bai et al., 2016). Participants were asked to indicate how often they engaged in each of the listed behavior using a 5-point scale (from 1 = strongly disagree to 5 = strongly agree). A sample item for CWB (α = 0.93) is “Spent time on tasks unrelated to work.”

#### The dark triad

The second variable of interest is the Dark Triad traits, i.e., psychopathy, narcissism, and Machiavellianism. The study uses a 12-item Dirty Dozen scale adapted from Jonason and Webster (2010). Items were rated on a 5-point Likert scale, ranging (from 1 = strongly disagree to 5 = strongly agree). Recent studies have used the Dirty Dozen scale as a measure of the Dark Triad, e.g., Atari and Chegeni (2017) and Rahafar et al. (2017). Sample items for psychopathy (α = 0.83), narcissism (α = 0.80), and Machiavellianism (α = 0.71) are “I tend to lack remorse,” “I tend to want others to admire me,” and “I tend to manipulate others to get my way,” respectively.

#### Perceptions of organizational politics

The third variable of interest is POPS, and a six-item scale adapted from the study of Kacmar and Ferris (1991) was used to measure POPS. The six-items represent general political behavior which is a core representative dimension of perceived organizational politics scale (Abbas et al., 2014). A five-point scale was used ranging from 1 = strongly disagree 5 = to strongly agree. The scale has been used as a POPS in previous studies to measure general political behavior (Byrne, 2005; Naseer et al., 2016). A sample item for POPS (α = 0.89) is “One group always gets their way.”

#### Political skills

Finally, a six-item scale was adapted from Ferris et al. (1999) for measuring political skills. A five-point response format from 1 = strongly disagree to = 5 strongly agree was used. A sample item for political skills (α = 0.87) includes “I am good at getting others to respond positively to me.”

Conway and Lance (2010) demonstrated that self-report survey is an appropriate method of the investigation if authors provide arguments for their decision and provide construct validity of the measures. In this regard, we employ self-report measures in the study because we believe that respondents of the present study are the best source of data regarding their beliefs. Moreover, we also demonstrate the construct validity of the measures used in the study.

## Results

The interpretation and analysis of the PLS model comprise of the two sets of linear equations. First, the measurement model states the relationship between constructs and its observed indicators. Second, the structural model indicates relationships between constructs (Henseler et al., 2016).

### The measurement model

Evaluating the measurement model involves the assessment of reliability and validity (Henseler et al., 2009). Results confirm that the measurement model met all requirements of model fit. First, all factor loadings are in general greater than 0.7, which satisfy the condition of reliability, as reported in Table 1. The study uses the item-trimming process in order to remove items with weak loadings values. In this vein, items Mach_4, POPS_6, and Pol_6 have been eliminated from the final analysis.

**Table 1:** The measurement model.

**Construct/dimension/indicator**	**VIF**	**Loadings**	**rho_A**	**Average variance**
				**Extracted (AVE)**
Psychopathy	2.037		0.852	0.648
Psy_1		0.767		
Psy_2		0.833		
Psy_3		0.817		
Psy_4		0.801		
Narcissism	2.354		0.822	0.614
Narc_1		0.757		
Narc_2		0.728		
Narc_3		0.830		
Narc_4		0.816		
Machiavellianism	2.067		0.723	0.623
Mach_1		0.811		
Mach_2		0.750		
Mach_3		0.806		
Mach_4		0.401^a^		
Perceptions of Organizational Politics	2.444		0.895	0.703
POPS_1		0.818		
POPS_2		0.869		
POPS_3		0.856		
POPS_4		0.805		
POPS_5		0.844		
POPS_6		0.393^a^		
Counterproductive Work Behavior			0.927	0.661
CWB_1		0.796		
CWB_2		0.805		
CWB_3		0.826		
CWB_4		0.789		
CWB_5		0.846		
CWB_6		0.849		
CWB_7		0.849		
CWB_8		0.738		
Political Skill			0.875	0.654
Pol_1		0.718		
Pol_2		0.841		
Pol_3		0.822		
Pol_4		0.813		
Pol_5		0.842		
Pol_6		0.415^a^		

a *The items were removed from the final version of the construct and not used in the structural model; All loadings are significant at 0.001 level (2-tailed); rho_A, Dijkstra-Henseler's rho indictors; VIF, Variance Inflation Factor*.

Second, the assessment of construct reliability uses Dijkstra-Henseler's rho indicators (Dijkstra and Henseler, 2015). As shown in Table 1, the minimum reliability value of 0.7 confirms the construct reliability of composite indicators (Henseler, 2017a). Third, the latent variables also meet the standard requirement of convergent validity because all values of their average variance extracted (AVE) surpasses 0.50 (Hair et al., 2016) (Table 1).

Finally, discriminant validity shows that the degree of a construct is truly distinct from other constructs by empirical standards. Following the recommendation of Fornell and Larcker (1981) and Henseler et al. (2015), the study follows Fornell-Larcker and heterotrait-monotrait (HTMT) criteria for discriminant validity, respectively. The Fornell-Larcker criterion is a common approach to assess the discriminant validity; it suggests that the square root of each construct's AVE should be higher than the correlation values with any other construct, as shown in Table 2 diagonal elements (bold). The values of heterotrait-monotrait (HTMT) are also below the threshold value of 0.85 in all cases (Table 2). Therefore, results confirm the existence of discriminant validity in the study.

**Table 2:** Measurement model: discriminant validity.

	**Fornell-Larcker Criterion**						**Heterotrait–monotrait ratio (HTMT)**					
	**CWB**	**Mach**	**Narc**	**POPS**	**Pol**	**Psy**	**CWB**	**Mach**	**Narc**	**POPS**	**Pol**	**Psy**
CWB	**0.813**											
Mach	0.714	**0.789**						0.846				
Narc	0.767	0.650	**0.784**					0.827	0.844			
POPS	0.774	0.653	0.686	**0.839**				0.848	0.806	0.777		
Pol	0.714	0.641	0.690	0.660	**0.808**			0.800	0.813	0.812	0.741	
Psy	0.723	0.580	0.637	0.655	0.630	**0.805**		0.765	0.710	0.730	0.716	0.716

*Psy, Psychopathy; Narc, Narcissism; Mach, Machiavellianism; POPS, Perceptions of organizational politics; Pol, Political Skills; CWB, Counterproductive work behavior*.

### The structural model

This study examines the issue of possible collinearity among predictor constructs. The values of VIF are below the threshold (<5), which confirms the absence of multicollinearity. Furthermore, to estimate the research model of the present work, we employ a bootstrapping procedure through 5,000 randomly drawn subsamples with replacement at 0.05% level of significance. The execution of bootstrapping provides a confidence interval and standard errors to assess the statistical significance of the variables of interest (Henseler et al., 2009; Hair et al., 2016). Table 3 presents the estimated values of path coefficients.

**Table 3:** Structural model results.

**Relationships**	**Model 1**	**Model 2**	**Model 3**	**Model 4**	***f*^2^**	**Support**
		$R_{\text{POPS}}^{2}$ = 0.59/$Q_{\text{POPS}}^{2}$ = 0.38	$R_{\text{POPS}}^{2}$ = 0.61	$R_{\text{POPS}}^{2}$ = 0.62		
	$R_{\text{CWB}}^{2}$ = 0.74/Q^2^ = 0.45	$R_{\text{CWB}}^{2}$ = 0.76/$Q_{\text{CWB}}^{2}$ = 0.46	$R_{\text{CWB}}^{2}$ = 0.76	$R_{\text{CWB}}^{2}$ = 0.76		
H1-a: Psy → CWB	(c_1_) 0.30^***^ (5.30) [0.18; 0.41]	(${\text{c}}_{1}^{\prime }$) 0.23^***^ (3.51) [0.10; 0.35]	(${\text{c}}_{1}^{\prime }$) 0.23^***^ (3.62) [0.09; 0.35]	(${\text{c}}_{1}^{\prime }$) 0.23^***^ (3.62) [0.09; 0.35]		Yes
H1-b: Narc → CWB	(c_2_) 0.40^***^ (7.51) [0.30; 0.51]	(${\text{c}}_{2}^{\prime }$) 0.29^***^ (4.97) [0.17; 0.40]	(${\text{c}}_{2}^{\prime }$) 0.29^***^ (4.95) [0.17; 0.40]	(${\text{c}}_{2}^{\prime }$) 0.29^***^ (4.87) [0.17; 0.40]		Yes
H1-c: Mac → CWB	(c_3_) 0.29^***^ (4.49) [0.15; 0.40]	(${\text{c}}_{3}^{\prime }$) 0.20^***^ (3.19) [0.07; 0.31]	(${\text{c}}_{3}^{\prime }$) 0.20^***^ (3.14) [0.07; 0.32]	(${\text{c}}_{3}^{\prime }$) 0.20^***^ (3.18) [0.07; 0.32]		Yes
Psyco → POPS = a_1_		0.29^***^ (3.41) [0.11; 0.45]	0.25^***^ (2.74) [0.06; 0.41]	0.26^***^ (2.95) [0.08; 0.42]		
Narc → POPS = a_2_		0.32^***^ (3.92) [0.15; 0.48]	0.26^***^ (2.82) [0.08; 0.43]	0.17^*^ (1.88) [−0.01; 0.35]		
Mac → POPS = a_3_		0.27^***^ (3.52) [0.12; 0.43]	0.23^***^ (2.85) [0.06; 0.38]	0.21^***^ (3.11) [0.08; 0.34]		
POPS → CWB = b		0.30^***^ (4.35) [0.17; 0.43]	0.30^***^ (4.46) [0.17; 0.43]	0.30^***^ (4.47) [0.17; 0.43]		
Pol → POPS = a_4_			0.18^**^ (2.58) [0.05; 0.32]	0.23^***^ (2.64) [0.06; 0.39]		
H3-a: Psy × Pol → POPS = a_5_				0.102^ns^ (1.118) [−0.08; 0.28]	0.006	No
H3-b: Narc × Pol → POPS = a_6_				0.187^**^ (2.488)[0.04; 0.34]	0.03	Yes
H3-c: Mac × Pol → POPS = a_7_				0.149^*^ (1.938) [0.002; 0.30]	0.01	Yes

*Psy, Psychopathy; Narc, Narcissism; Mac, Machiavellianism; CWB, Counterproductive Work behavior; POPS, Perceptions of Organizational Politics; Pol, Political Skill*.

*t-values in parentheses, Bootstrapping 95% confidence intervals bias corrected in square brackets (based on n = 5,000 subsamples)*.

ns, not significant ^***^p < 0.01, ^**^p < 0.05, ^*^p < 0.1, [based on t_(4999)_, two-tailed test].

**Figure 1:**
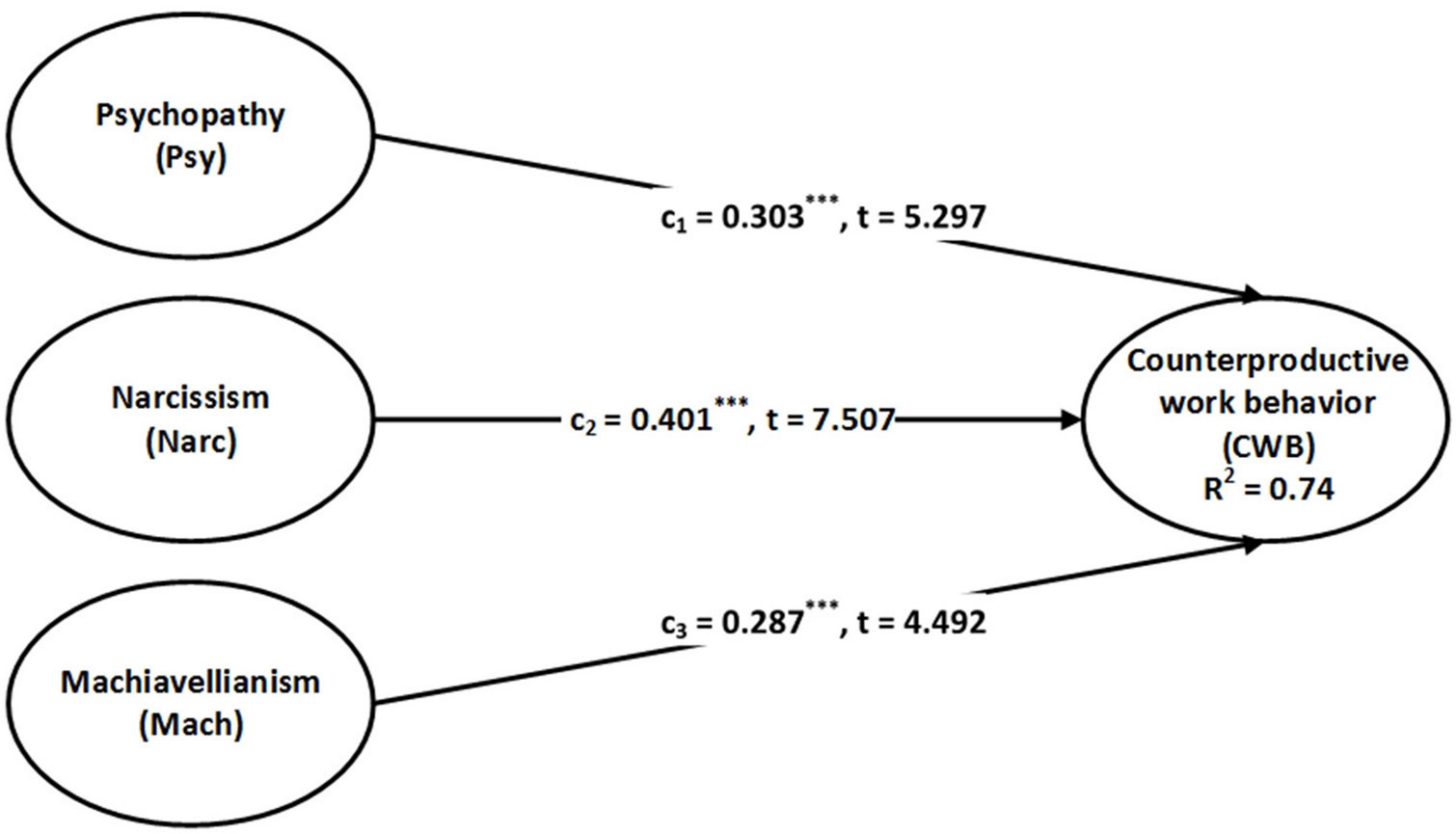
Model with total effect (Model 1).

**Figure 2:**
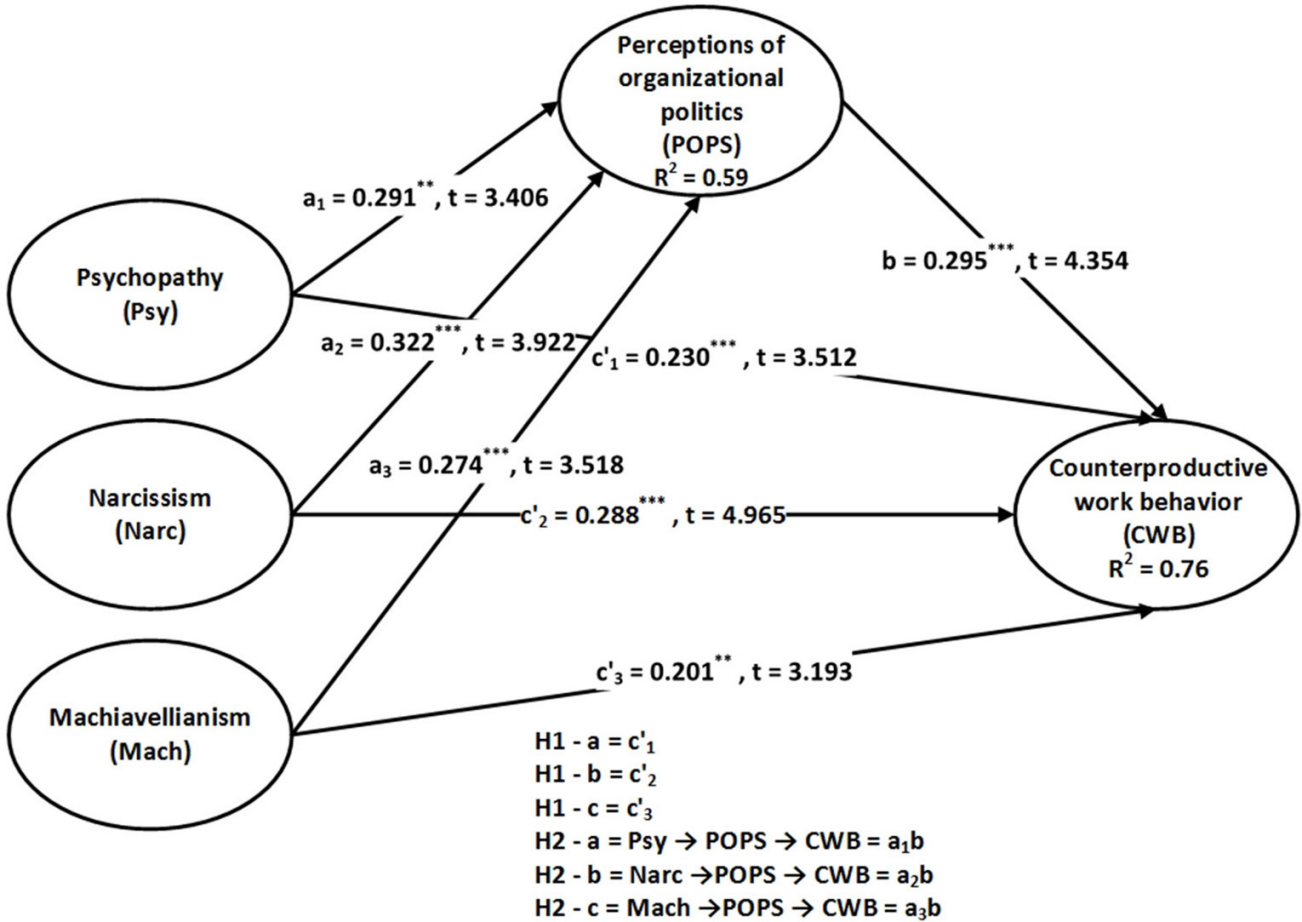
Model with an indirect effect (Model 2).

The process of the structural equation model and PROCESS are completed in four steps. First, we determine the total effect to check the direct influence of the Dark Triad on CWB. Table 3 presents the structural assessment of the variables of interest in four models. In the case of Model 1, results show the significant total effects of psychopathy, narcissism, and Machiavellianism on CWB, also shown in Figure 1. Model 2 and Figure 2 demonstrate that after including POPS as a mediator, psychopathy, narcissism, and Machiavellianism still have significant direct effects on CWB. These facts support H1-a, H1-b, and H1-c, respectively. Furthermore, the direct effects of a_1_, a_2_, a_3_, and b, i.e., the Dark Triad to POPS and POPS to CWB, are also significant. Here it is worthy to note that the direct effect decreases after including the mediator. Hence, the reduction observed in the direct effects ${\text{c}}_{1}^{\prime }$, ${\text{c}}_{2}^{\prime }$, ${\text{c}}_{3}^{\prime }$, and the significance of the path coefficients a_1_, a_2_, a_3_, and b imply the presence of indirect effects of the Dark Triad on CWB via POPS as a mediator. Nevertheless, the essential requirement is to test whether indirect effects a_1_b, a_2_b, and a_3_b are significant (Hayes, 2013).

In the second step, we employ a non-parametric bootstrapping procedure to investigate the significance of indirect effects (Preacher and Hayes, 2008; Nitzl et al., 2016). The 95% confidence intervals are generated by 5,000 resamples for the mediator. The results in Table 4 show that the indirect effects a_1_b, a_2_b, and a_3_b are also significant. These results support H2-a, H2-b, and H2-c, respectively. In addition, the study determines the type and magnitude of the mediation. In this regard, the study assumes a partial mediation of the POPS in the relationship between the Dark Triad and CWB as the direct effects and indirect effects are both significant. Furthermore, the study observes complementary partial mediation because the product of a_1_ x b x ${\text{c}}_{1}^{\prime }$, a_2_ x b x ${\text{c}}_{2,}^{\prime }$ and a_3_ x b x ${\text{c}}_{3}^{\prime }$ is positive. It shows that a portion of the effect of the Dark Triad on CWB is mediated through POPS, whereas the Dark Triad still explains a part of CWB that is independent of POPS. Accordingly, this study employs variance accounted for (VAF) to calculate the magnitude of mediation. VAF determines the extent to which the indirect effects a_1_b, a_2_b, and a_3_b explain about the total effects c_1_, c_2_, and c_3_, respectively. The values of VAF in Table 4 are within the range of 20–80% that demonstrates the partial mediation (Nitzl et al., 2016).

**Table 4:** Summary of mediating effect tests.

	**Total effect on CWB (Model 1)**				**Direct effects on CWB (Model 2)**					**Indirect effects on CWB (Model)**						
	**Path**	***t***	**BCCI**			**Path**	***t***	**BCCI**			**Point estimate**	***t***	**BCCI**		**Sig**	**VAF**
			**Lower**	**Upper**				**Lower**	**Upper**				**Lower**	**Upper**		
Psy (c_1_)	0.30^***^	8.30	0.18	0.41	H1-a = c'_1_	0.23^***^	3.51	0.10	0.35	H2-a = a_1_b_1_ (via POPS)	0.086^***^	2.94	0.039	0.156	Yes	28.67%
Narc (c_2_)	0.40^***^	7.51	0.30	0.51	H1-b = c'_2_	0.29^***^	4.97	0.17	0.40	H2-b = a_2_b_1_ (via POPS)	0.095^***^	2.84	0.042	0.184	Yes	23.75%
Mach (c_3_)	0.29^***^	4.49	0.15	0.40	H1-c = c'_3_	0.20^***^	3.19	0.07	0.31	H2-c = a_3_b_1_ (via POPS)	0.081^**^	2.47	0.031	0.162	Yes	27.93%

*Psy, Psychopathy; Narc, Narcissism; Mach, Machiavellianism; POPS, Perceptions of organizational politics; CWB, Counterproductive work behavior; BCCI, Bias corrected confidence interval. Bootstrapping based on n = 5,000 subsamples*.

*VAF, Variance accounted for. VAF > 80% shows full mediation, 20% ≤ VAF ≥ 80% indicates partial mediation while VAF < 20% represents no mediation*.

*** p < 0.01,

** *p < 0.05 [based on t_(4999)_, two-tailed test]*.

**Figure 3:**
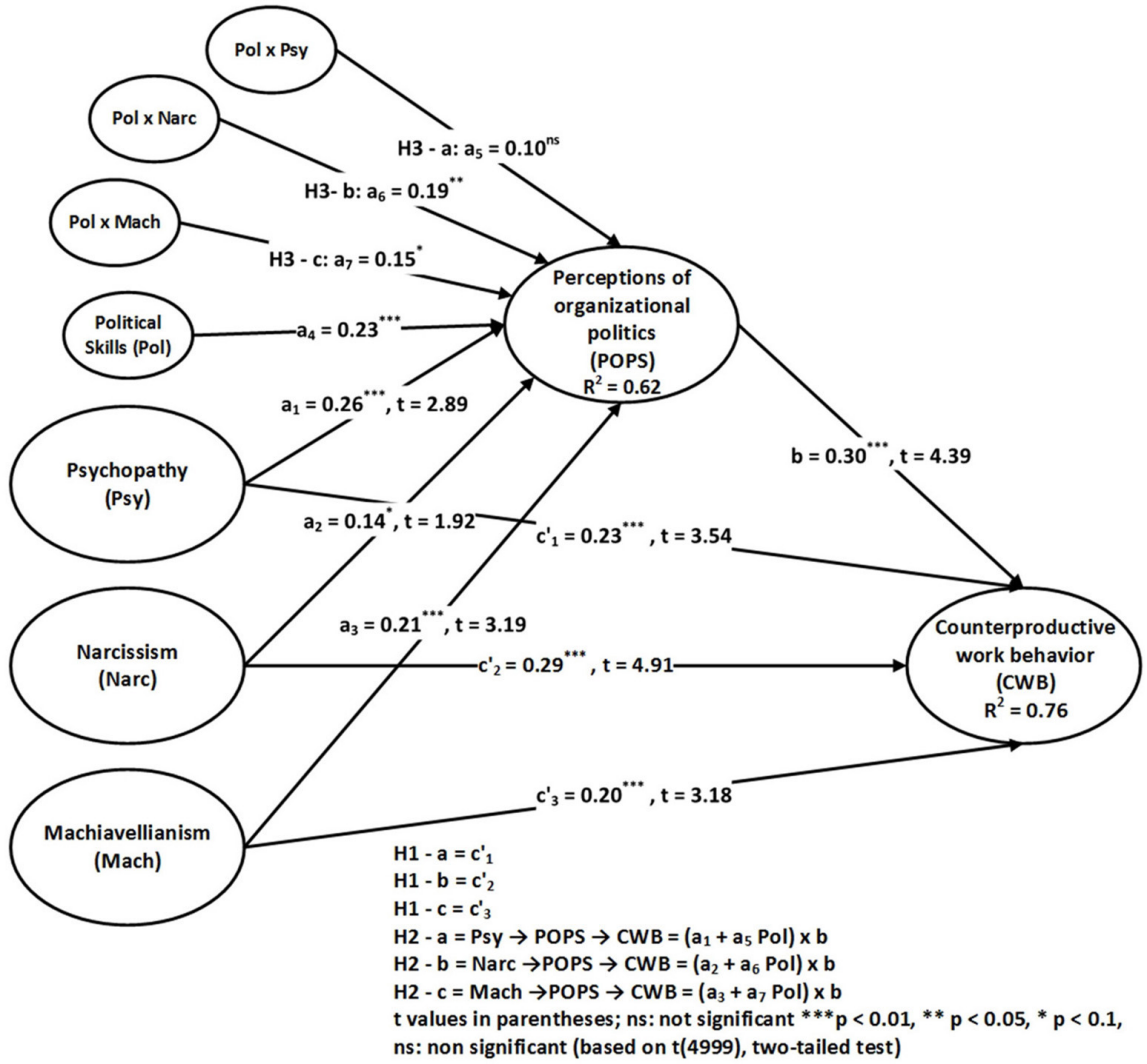
Model with conditional indirect effect (Model 4).

In the third step, we evaluate the moderation hypotheses H3-a, H3-b, and H3-c of political skills on the path between the Dark Triad and POPS by employing the product indicator technique. Model 3 adds (Pol), and Model 4 includes the interaction terms Pol x Psy, Pol x Narc, and Pol x Mach. As shown in Figure 3, Table 3, and Model 4, the results support H3-b and H3-c; however, they do not support H3-a. Furthermore, the overall effect size for a_6_ and a_7_ attain an f^2^ value of 0.03 and 0.01, respectively, whereas effect size for a_5_ is not up to the threshold level. It is important to recognize that a small f^2^ does not indicate a negligible effect. “If there is a likelihood of occurrence for the extreme moderating conditions and the resulting beta changes are meaningful, then it is important to take these situations into account” (Limayem et al., 2001; Ali and Park, 2016). However, Kenny (2016) recommended that 0.005, 0.01, and 0.025 constitute realistic standards for small, medium, and large effect sizes, respectively. In this scenario, a_6_ and a_7_ attain medium and large effect size, respectively. According to Hayes (2013), the significance of the moderating effect in combination with the indirect effects establishes a foundation to assess the conditional process model. The effects of psychopathy, narcissism, and Machiavellianism on POPS are contingent on political skills (Pol); therefore, the indirect effects of psychopathy, narcissism, and Machiavellianism influence CWB. According to Hayes (2013), such indirect effects are the product of the conditional effects of psychopathy, narcissism, and Machiavellianism on POPS (a_1_ + a_5_Pol), (a_2_ + a_6_Pol), (a_3_ + a_7_Pol), respectively, and the unconditional effect of POPS on CWB (b). The conditional indirect effects are (a_1_ + a_5_Pol) x b, (a_2_ + a_6_Pol) x b, (a_3_ + a_7_Pol) x b, respectively.

Finally, we apply the PROCESS macro developed by Hayes (2013) to calculate the conditional indirect effects to confirm the moderated-mediation^2^. This study takes latent variable scores from SmartPLS 3.2.6 as an input, PROCESS-generated estimates, and a bias-corrected 95% bootstrap CI for the indirect effects at different values of the moderator (Pol). Table 5A represents that the indirect effects of psychopathy, narcissism, and Machiavellianism on CWB through POPS, which are consistently positive and increase as the value of the moderator rises. The 95% bias-corrected bootstrap confidence intervals (CI) for the conditional indirect effects are above zero for medium-to-high values of political skills. If the value of political skills is small, the point estimate indicates that the indirect effect is still positive, while the confidence interval is not different from zero (includes zero). Therefore, POPS partially mediates psychopathy, narcissism, and Machiavellianism effects on CWB, except when the values of political skills are small. In the case of lower values of political skills, the indirect effects are insignificant. In the end, Table 5B reports an index of moderated mediation for psychopathy, narcissism, and Machiavellianism, which is also significant for narcissism and Machiavellianism since zero falls outside of the CI. While in the case of psychopathy, it is not significant because CI includes zero (Hayes, 2013).

**Table 5A:** Conditional indirect effect analyses: Conditional indirect effects of Psychopathy, Narcissism, and Machiavellianism on Counterproductive work behavior (CWB) through Perceptions of organizational politics (POPS) at values of Political Skills as moderator.

**Mediator**	**Political Skills**	**Indirect effect**	**Boot SE**	**BCCI**	
				**Lower**	**Upper**
POPS _(P)_	−0.867	0.138	0.091	−0.041	0.311
POPS _(P)_	0.000	0.189	0.064	0.071	0.322
POPS _(P)_	0.867	0.241	0.062	0.129	0.377
POPS _(N)_	−0.867	0.059	0.069	−0.071	0.201
POPS _(N)_	0.000	0.147	0.052	0.053	0.258
POPS _(N)_	0.867	0.235	0.056	0.136	0.354
POPS _(M)_	−0.867	0.121	0.065	−0.014	0.244
POPS _(M)_	0.000	0.195	0.052	0.096	0.305
POPS _(M)_	0.867	0.269	0.063	0.151	0.404

*Values for Political Skills (moderator) are mean and plus/minus one standard deviation (SD) from the mean*.

**Table 5B:** Conditional indirect effect analyses: Index of moderated mediation.

**Mediator**	**Index**	**SE (Boot)**	**Bias-corrected bootstrap 95% confidence interval**	
			**Lower**	**Upper**
POPS _(P)_	0.060	0.052	−0.033	0.172
POPS _(N)_	0.102	0.041	0.030	0.191
POPS _(M)_	0.085	0.043	0.004	0.175

*POPS, Perceptions of Organizational Politics, (P), (N), and (M) represents Psychopathy, Narcissism, and Machiavellianism respectively*.

*Bootstrapping based on n = 5,000 subsamples*.

## Discussion

The studies in recent decades have acknowledged that examination of the dark side of the organization is equally important as examination of the bright side of the organization (Schyns, 2015). The aspects of the Dark Triad personalities of employees in the context of the dark side of the organization have not been investigated sufficiently. However, the recent academic wave of the dark side of the organization has brought this concept from margin to surface (Harms and Spain, 2015). This study contributes to the growing body of knowledge by digging more into the dark side of organizational research by investigating the relationship between the Dark Triad and CWBs.

The present study focuses on the relationship between the Dark Triad and CWB by examining the mediating and moderating roles of POPS and political skill, respectively, in the nexus of the Dark Triad and CWB. Numerous studies theoretically tested the relationship between the Dark Triad and CWBs. However, the question remains unsolved whether the effect of the Dark Triad on CWB is more a direct effect or an indirect effect brought about through other organizational factors. Moreover, there is a need to determine the potential moderating role of political skills on the relationship between the Dark Triad personalities and CWB.

First, our empirical findings reveal a direct relationship between the Dark Triad and CWB, a nexus that could not be clarified in the previous literature. Second, turning to the mediating effect, this study also provides evidence for the existence of an indirect effect of the Dark Triad on CWB through POPS. Our results support the direct link between the Dark Triad and POPS. The results are in line with prior literature, which shows that personalities are important antecedents of POPS (Ferris et al., 1989). The study of Valle and Perrewe (2000) also provided sufficient support for the link between the Dark Triad and POPS. In the same way, our results find adequate support for the second-half of the mediation, i.e., the POPS–CWB link. Results are also consistent with the findings of Cohen and Diamant (2017), who reported a positive relationship between POPS and CWB. Results reveal interesting facts that the Dark Triad influences CWB more through POPS than directly. Hence, we infer that the Dark Triad traits affect CWB enhancement concerning the extent that the Dark Triad perceives a high level of politics.

Third, this study gives an in-depth understanding of the politically skilled repercussions of the Dark Triad–POPS link. Indeed, findings support the Dark Triad and POPS link; circumstances to do with political skills affect this mediation. As mentioned above, the mediating role is positive and significant. Findings further reveal that the mediating relationship may get stronger when the Dark Triad personalities have a high level of political skills. As per the hypotheses of the study, political skills positively moderate the Dark Triad and POPS link. Results of the moderating effect reveal that the Dark Triad positively affects POPS, exhibiting that POPS outcomes get much stronger when the Dark Triad personalities possess high political skills, particularly in the case of narcissism and Machiavellianism. These results support H3-b and H3-c. Nevertheless, H3-a, in this case, could not find sufficient support. The findings are justified because the level of political skills varies from person to person, even among those who score high in the Dark Triad construct (Cohen, 2016). It leads to the conclusion that psychopathy, in our case, possesses low-level skills as compared to the rest of the Dark Triad personalities. Finally, the Dark Triad and POPS provide reasonable predictions of the CWB variable.

This study presents some significant academic contributions, extending the relationship between the Dark Triad figures and CWBs, by investigating the mediating effect of POPS between the Dark Triad and CWBs and the moderating influence of political skills on the link between the Dark Triad and POPS. First, the findings of the study provide sufficient support to fill the existing gap concerning the direct or indirect effect of the Dark Triad on CWB. As proposed, the study concludes that POPS partially mediates the Dark Triad and CWB link. Empirical findings reveal important practical implications for the hospitality industry. Mediating analysis of the study suggests that possible CWB, due to the Dark Triad, can be minimized by controlling POPS. The Dark Triad personalities perceive organizational politics as an advantage in manipulating a situation, which leads to the possibility of high CWBs. Hence, managers should take notice in an effort to mitigate the negative impacts linked with the Dark Triad. One way to reduce the intensity of such behaviors is to develop an ethical culture and implement regular training sessions that promote ethical values at the workplace. In the same way, managers should improve their control of the environment by developing a visible and transparent system. As mentioned by Kaptein (2008), transparency has the potential to expose unethical practices and increases the possibility of getting caught.

Second, the results also contribute to the existing literature that the presence of political skills among the Dark Triad may lead to an increase in CWB in an organization. The findings provide evidence that, acting as a moderator variable, political skills positively moderate the effect on the Dark Triad and POPS link. However, in the case of a psychopathy and POPS link, the moderating effect, surprisingly, contrasts with the proposed hypothesis. This finding is theoretically quite interesting, nonetheless, because it provides an explanation into the idea that the level of political skill varies even among those who score high on the Dark Triad constructs. The theoretical model and empirical findings of the study suggest that managers in the hospitality sector should consider the effects of the personalities and political skills of its employees. In this manner, the recruitment process can play a crucial role in identifying personality traits and political skills. Different instruments, such as questionnaires and scenarios simulation, can be utilized to determine personalities and political skills. It is important to note that it seems unfair to screen out any of the Dark Triad personalities or those high in political skills. Managers and organizations can utilize their unique traits and competencies fruitfully. Organizations may organize training programs, counseling sessions, and mentoring facilities to get favorable outcomes from these traits.

### Limitations and future research

Our research presents several limitations, which provide an avenue for future studies. First, the data have been collected only from a single sector (hospitality) and country (China). Therefore, future research should consider other industries and cultures for more generalized results. Second, our study focuses on POPS as a mediator in the relationship between the Dark Triad and political skills as a moderator in the relationship between the Dark Triad and POPS. An auspicious future direction could include exploring additional mediating and moderating factors that could clarify the Dark Triad and CWB relationships. Third, the study has used the Dirty Dozen scale of Jonason and Webster (2010) to measure the Dark Triad. Nevertheless, brief measures of personality traits have both costs and benefits. They are costly because they may be limited in detecting comprehensive attributes. They may be beneficial by eliminating unnecessary items (Czarna et al., 2016). Therefore, future research should employ other measures like SD3 of Jones and Paulhus (2014) or full measure of the Dark Triad. Fourth, our study is based on cross-sectional research design; future research should aim to apply longitudinal designs that may explore causal effects between these constructs (Karim et al., 2013). Finally, the sample size of the current study is relatively small. Future researchers should consider this limitation and use larger sample size.

## Conclusion

The present work develops and empirically examines a conceptual model that explains the role POPS plays as a mediator between the relationship between the Dark Triad and CWB. Moreover, the present work highlights the moderating role of political skills on the relationship between the Dark Triad and the POPS. A questionnaire survey has been used to collect data from the hospitality sector of China. The PLS structural equation modeling and the PROCESS tool have been employed to analyze the data. Our findings reveal some interesting facts that the previous studies could not address in the relationship between the Dark Triad and CWB. We conclude that POPS is an important mediator in the relationship between the Dark Triad and CWB. Furthermore, this mediating effect gets stronger when the Dark Triad possesses high political skills.

## Author contributions

MAB developed the main idea of the study, carried out empirical studies, participated in the sequence alignment, and drafted the manuscript. FM refined the idea and supervised the study, and participated in its design and coordination and helped to draft the manuscript. ZX helped out in data collection. IC-C provided valuable support in statistical analysis. D refined the flow and logic of the manuscript. MWB refined the language and provided valuable comments. All authors read and approved the final manuscript.

### Conflict of interest statement

The authors declare that the research was conducted in the absence of any commercial or financial relationships that could be construed as a potential conflict of interest.

## Footnotes

^1^Psychopathy, Narcissism, and Machiavellianism.

^2^Moderated mediation occurs when a moderator variable interacts with a mediator variable such that the value of the indirect effect changes depending on the value of the moderator variable (Joseph F Hair et al., 2016, P. 292)
